# The human protein atlas—Integrated omics for single cell mapping of the human proteome

**DOI:** 10.1002/pro.4562

**Published:** 2023-02-01

**Authors:** Andreas Digre, Cecilia Lindskog

**Affiliations:** ^1^ Department of Immunology, Genetics and Pathology Uppsala University Uppsala Sweden

**Keywords:** antibodies, data integration, omics, open‐access, proteomics, single‐cell, spatial

## Abstract

Studying the spatial distribution of proteins provides the basis for understanding the biology, molecular repertoire, and architecture of every human cell. The Human Protein Atlas (HPA) has grown into one of the world's largest biological databases, and in the most recent version, a major update of the structure of the database was performed. The data has now been organized into 10 different comprehensive sections, each summarizing different aspects of the human proteome and the protein‐coding genes. In particular, large datasets with information on the single cell type level have been integrated, refining the tissue and cell type specificity and detailing the expression in cell states with an increased resolution. The multi‐modal data constitute an important resource for both basic and translational science, and hold promise for integration with novel emerging technologies at the protein and RNA level.

## 1 INTRODUCTION

Recently, different human mapping efforts have provided an overview of gene and protein expression across a wide range of tissues and organs. Such initiatives include the GTEx (Keen & Moore, 2015) and FANTOM5 (Yu et al., 2015) consortia based on bulk RNA sequencing, and the Human Proteome Project (HPP), generating a high‐stringency map of human proteins using mass spectrometry (Adhikari et al., 2020). In parallel, a decade of development of the single cell RNA sequencing (scRNAseq) method has opened up the possibility to solve the complexity of the human body at completely new levels. Body‐wide efforts focusing on single‐cell transcriptomics include the Human Cell Atlas (HCA) (Regev et al., 2017), the Chan Zuckerberg Initiative (CZI) (Tabula Sapiens et al., 2022), the Human BioMolecular Atlas Program (HuBMAP) (Consortium, H, 2019) (funded by the National Institute of Health [NIH]), and the Human Cell Landscape (Han et al., 2020). Few previous efforts however focused on integration of various approaches, and studies that involve validating transcriptomic findings at the protein level are rare.

**FIGURE 1:**
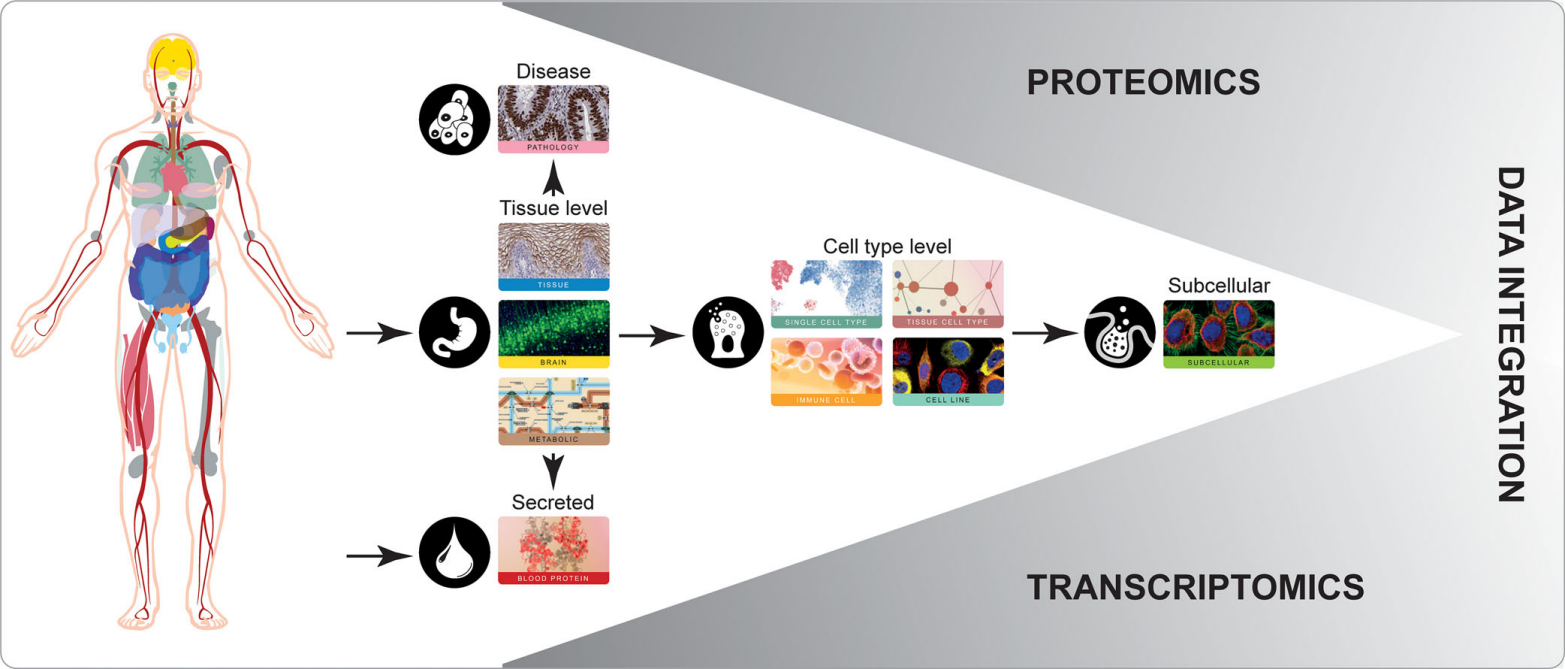
Overview of the content of the Human Protein Atlas database. The open‐access resource contains comprehensive information on different aspects of the human proteome, divided into 10 separate sections. By integrating proteomics with transcriptomics, the human protein‐coding genes can be analyzed for specificity at the tissue, cellular and subcellular level, as well as during disease or in human blood

In the Human Protein Atlas (HPA) project, a combination of transcriptomics and antibody‐based proteomics is used to map all human proteins at a single‐cell and spatial resolution (Uhlen et al., 2015). All data is made publically available in the open‐access knowledge resource http://www.proteinatlas.org, that has grown into one of the world's most visited biological databases. In version 21 that was released in November 2021, a major update was launched introducing 10 different sections instead of the previously six (Digre & Lindskog, 2021), covering various aspects of human protein‐coding genes (Figure 1). New sections include Single Cell Type, Tissue Cell Type, Cell Line, and Blood Proteins. A new way of classifying genes was also introduced in version 21, by which all genes were clustered based on expression across the different tissues/cell types, followed by annotation of each cluster to assign main properties according to common features in terms of overall function, location, and specificity. This novel classification covers 87 tissue gene clusters, 68 single cell type gene clusters, 52 immune cell gene clusters and 44 cell line gene clusters.

With the new data and organization of the database, the HPA gives the users a possibility to explore proteins from new angles and with a higher resolution. Here, we provide an overview of the recent updates and the structure of the HPA database, with examples showing the utility of the different datasets and how these can be integrated for a comprehensive view of human proteins at different levels.

## 2 INTEGRATED OMICS FOR INCREASING THE SINGLE CELL RESOLUTION

Transcriptomics methods are superior in terms of gene coverage, while mass spectrometry‐based proteomics methods exceed in dynamic range (Wang et al., 2019). Furthermore, proteomics can generate important information on temporal expression or to which extent proteins are modified, activated or repressed, including post‐translational modifications and various splice variants, information that cannot be retrieved by transcriptomics alone (Hikmet et al., 2020). One of the disadvantages of massive parallel sequencing of RNA and mass spectrometry is however the loss of transcript or protein location in relation to the tissue geography. Antibody‐based proteomics is to date the main method for determining spatio‐temporal expression at a single cell or subcellular level due to higher pixel resolution, but at the price of less quantitative measurements. Integrating data across disciplines aids in translating the findings into biologically relevant knowledge. In the HPA, multiple technologies for mRNA and protein detection are combined to build a multi‐dimensional spatio‐temporal map of the human body. In the following sections, we describe the most recent updates of the transcriptomics and proteomics datasets in the HPA, and how these were integrated to increase the resolution at a single cell level.

### 2.1 Single cell type—integration with single cell transcriptomics

Massive parallel sequencing of RNA using single cell preparations of tissues followed by computational cluster analysis has become a successful recipe used by researchers worldwide to study the human cellular components at healthy and diseased states. In the HPA, the effort to compile multiple publically available single cell transcriptomics datasets into a single body‐wide expression map of all protein‐coding genes was accomplished in two steps. In an initial effort, quality controlled datasets representing 13 tissues and PBMCs were compiled (Karlsson et al., 2021). Briefly, raw expression data from single cells of each tissue sample were grouped into clusters based on their expression across genes. The main cell type of each cluster was identified through manual annotation based on the mRNA expression of a large set of well‐known cell type markers and IHC‐based expression data. In order to handle genes with low expression levels, expression data within cell type clusters were pooled, finally generating normalized transcript per million (nTPM) for each gene and cell type. The processed data was added to version 20 of the HPA in the form of a *Single Cell Type* section (www.proteinatlas.org/humanproteome/single+cell+type). In version 21, 12 new tissues were added using the same data processing pipeline, increasing the total coverage to 25 tissues and PBMCs, giving rise to 444 individual cell type clusters (Karlsson et al., 2022). Cell type clusters within a tissue that were annotated with the same name, as well as cell types found in multiple tissues, such as immune cells, connective tissue cells, and endothelial cells, were merged. Altogether, this resulted in 76 main single cell types, corresponding to the major cell types in the human body. For the purpose of simplifying the interpretation of the data, all cell types were also organized into 15 different color‐coded cell type groups based on common functional features, for example, neuronal cells, germ cells, and various types of epithelial cells.

Classification of genes according to specificity of expression within the human body has been implemented previously in the HPA for bulk RNA‐seq data in order to highlight genes with interesting expression patterns across tissues (Ahlen et al., 2015). The same strategy was utilized to classify the specificity of gene expression across cell types (Karlsson et al., 2021, 2022). The specificity describes to which degree the expression of a gene is elevated among one or a few cell types compared to all other cell types. Elevated expression is divided into three different degrees of specificity, including (a) *enriched expression*, where a gene has at least a four‐fold higher expression in a cell type compared any other cell types, (b) *group enriched*, where a gene has at least a four‐fold higher expression in a group of 2–10 cell types compared to any other cell type, or (c) *enhanced*, where a gene has at least a four‐fold higher expression in 1–10 cell types compared to the mean expression of the other cell types. Most genes (*n* = 15,317, 76%) were classified as elevated in one or more cell types (Karlsson et al., 2022). This is a marked increase compared to the bulk RNA‐seq data (*n* = 10,986, 55%), which is expected since many of the analyzed cell types are found across many tissues. Among the elevated genes, 1908 genes (12%) were enriched in a single cell type, 2799 genes were group enriched, and 10,610 genes (69%), about half of all protein‐coding genes, were classified as enhanced in up to 10 cell types.

Expression levels (nTPM) of all protein‐coding genes in all cell types and single cell clusters, cell type specificity classifications, single cell and gene cluster visualizations, and integrated IHC images are all publically available at the Single Cell Type section of the HPA web portal (http://www.proteinatlas.org). The gene expression of each cell type and all associated cell type clusters can also be explored through the Single Cell Type section pages that include brief knowledge chapters sorted into 15 cell type groups, including comprehensive examples of cell type‐elevated genes. In addition, the Single Cell Type section pages include a dendrogram that shows the relationship between the different cell types, a gene cluster UMAP plot highlighting the relationship between genes across cell types as well as a summary of all cell type elevated genes. Further, the Single Cell Type data is integrated into the gene search result pages, giving the visitor the possibility to access all information from a gene‐specific perspective.

### 2.2 Tissue cell type—deconvolution of bulk RNA‐seq data

Parallel to the development and emerging role of single cell transcriptomics in the field of omics during the last decade, a complementary method has emerged for single cell type analysis. Based on computational deconvolution and integrated network analysis of bulk RNA‐seq data, a prediction of cell type specificity can be made for each gene based on the expression (Butler et al., 2016; Dusart et al., 2019). The method takes advantage of the fact that there are genes that are particularly expressed by a single cell type in a regular manner, so‐called reference genes, and thus reflect the quantity of that cell type in a tissue sample. After careful selection of a set of reference genes for each cell type, the expression levels of the reference genes are compared to the expression levels of all other genes across all tissue samples to determine the degree of correlation (0–1), thus creating surrogate measurements of the correlation between specific cell types and all protein‐coding genes. The cell type correlation data of all protein‐coding genes in 14 tissues has been added to the HPA website in the *Tissue Cell Type* section, constituting a useful complement to the bulk and single cell RNA‐seq data found in the Tissue and Single Cell Type sections of HPA, respectively. In an effort to profile highly cell type‐specific genes, the correlation data has been classified into specificity categories, where genes with large differences in correlation scores between one cell type and all other cell types within a tissue have been classified as enriched in that cell type and tissue. The enriched cell type‐specific genes can be explored either through a summary list and tissue‐based cell type profiles accompanied with in‐house generated IHC images in the Tissue Cell Type section pages, or by including the Tissue Cell Type specificity categories in advanced filtered searches to identify enriched genes supported by data from additional sections in the HPA. In addition to the cell type profiles organized into 14 different tissue pages, there are also cell type profiles for eight different “core cell types”, highlighting cell types found in all or most of the profiled tissues, including endothelial cells, smooth muscle cells, fibroblasts and five different types of immune cells.

Since the deconvolution method generates correlation scores based on reference genes without any estimate on quantities, it is seen as a complement to techniques that are able to quantify gene expression. The established Tissue Cell Type platform in the HPA provides a useful complementary tool to support data from the other sections and identify important cell type‐specific genes missed by other methods. The deconvolution analysis is able to avoid key issues associated with scRNA‐seq and antibody‐based analysis. While scRNA‐seq of tissue samples enables large‐scale high‐resolution analysis of the transcriptome, the method is limited by the need for tissue separation into its single cell components, a tricky procedure that results in the use of few tissue replicates, loss of certain types of cells that are fragile or hard to separate (e.g., Sertoli cells in testis), as well as an obvious risk of altering the expression profile of cells when they become detached from their environment (Chen et al., 2019; Dusart et al., 2019). One limitation of the deconvolution method is however that it is based on correlation with the expression of well‐known markers only. Thus, only previously identified cell types can be characterized, and the method is not suitable for defining novel subsets of cells and cell state‐specific expression patterns. Antibody‐based analysis of protein expression in tissues offers high‐resolution spatial localization of proteins, but struggles with varying degrees of specificity and off‐target binding, and has hence benefited greatly from supportive data generated by complementary methods (Sivertsson et al., 2020). In contrast to scRNA‐seq analysis, bulk RNA‐seq‐based deconvolution can utilize large numbers of freely available biological replicates for robust analysis of cells in their natural tissue environment. Deconvolution further allows for investigation into the expression specificity profile of low abundant and fragile cell types not previously characterized in the HPA by any of the previously used methods, such as podocytes of renal glomeruli, beta cells in pancreatic islets of Langerhans, gastric acid‐producing parietal and chief cells of the stomach, mast cells of the immune system, and several cell types of various cellular structures in the skin.

**FIGURE 2:**
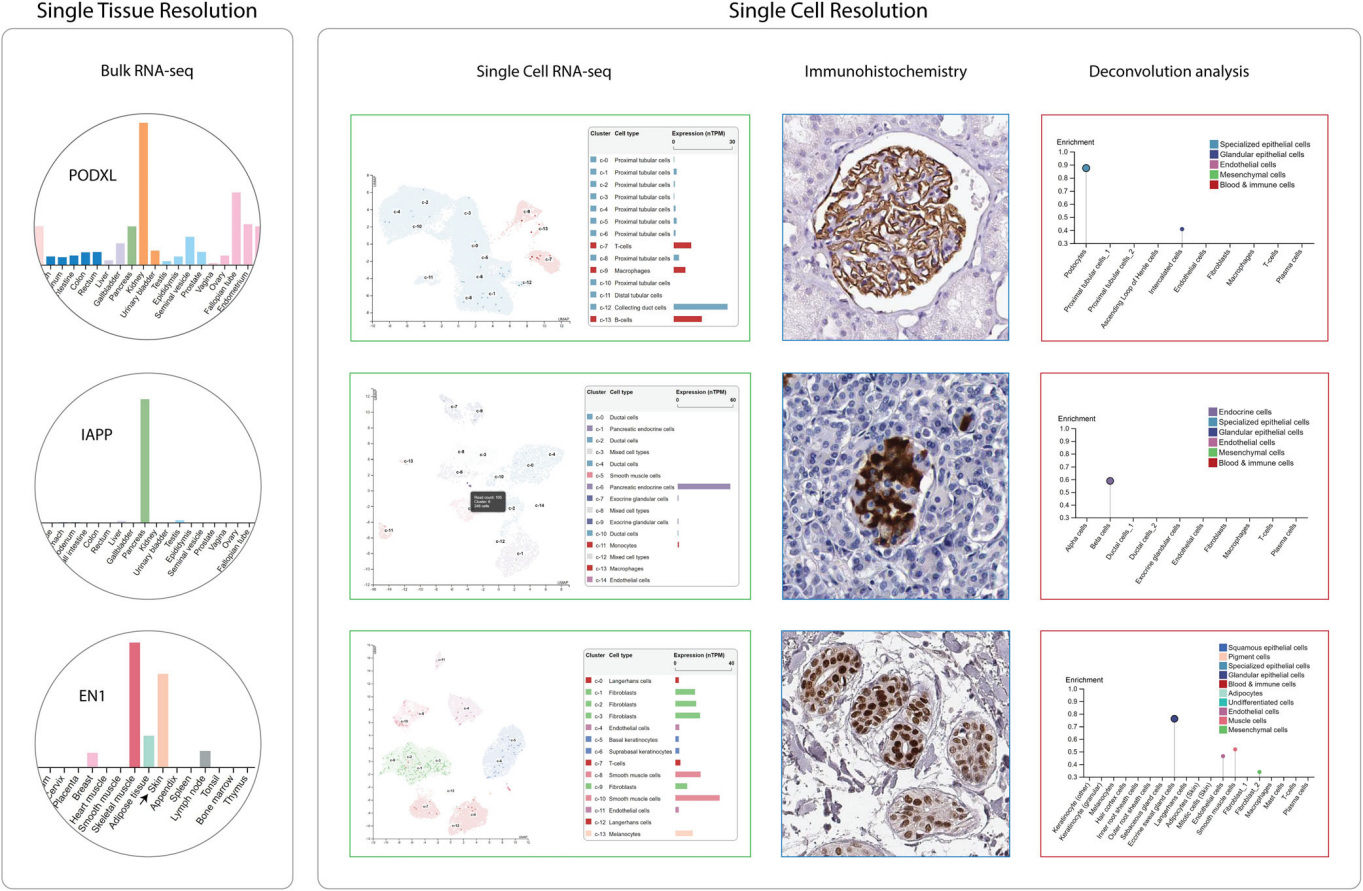
Integrated omics for increasing the single cell resolution. Representation of how the utilization of three types of expression data at single cell resolution (single cell RNA‐seq, immunohistochemistry and deconvolution analysis) enable increased understanding of the spatial expression compared to expression data at single tissue resolution (bulk RNA‐seq). Bulk RNA‐seq data (left panel) is presented as normalized transcripts per million (nTPM) in relevant tissues. Single cell resolution data (right panel) are shown for the relevant tissue for three different examples (PODXL/kidney, IAPP/pancreas and EN1/skin). Single cell RNA‐seq data is presented as tissue‐specific cell type cluster UMAP plots and bar charts. A mouse‐over window is shown in one of the cell type cluster UMAP plots (IAPP) listing cell‐associated read count, cluster number and the amount of cells in that cluster. Deconvolution analysis is represented by plots with enrichment scores for each cell type above cutoff (0.3). EN1, engrailed homeobox 1; IAPP, Islet amyloid polypeptide; PODXL, podocalyxin like

The benefit of integrating gross data at single tissue resolution with information from various methods at single cell resolution was evaluated by examining the specificity profiles of three of the “new” cell types at the Tissue Cell Type section: renal podocytes, pancreatic beta cells, and eccrine sweat gland cells of the skin. Podocytes are specialized epithelial cells that cover the capillaries of the kidney glomerulus, creating thin gaps between adjacent podocytes where only small molecules can pass through (Reiser & Altintas, 2016). Podocalyxin like (PODXL) is one of 43 genes classified as very highly enriched in podocytes (corr: 0.874) and is known in scientific literature to be expressed by podocytes to enable glomerular filtration (Refaeli et al., 2020). Through the employment of the bulk RNA‐seq data in the Tissue section, the expression of PODXL is shown to be localized to the kidney (Figure 2). However, to sort out the contribution from each of the different cell types within the kidney, expression data at single cell resolution is needed. While the kidney scRNA‐seq data that was included in version 21 of the Single Cell Type section lacks a podocyte cluster and only shows low level of expression in mainly collecting duct cells, IHC images show clear expression of PODXL in the outer podocyte layer of glomeruli, supported by the high correlation of expression between PODXL and the podocyte reference transcripts predicted by the deconvolution analysis in the Tissue Cell Type section.

Islet amyloid polypeptide (IAPP) is a well‐characterized protein hormone that is known to be produced by beta cells of islets of Langerhans and secreted into the blood together with insulin in order to regulate the levels of glucose in the blood. IAPP is classified as very highly enriched in beta cells (corr: 0.588) within the pancreas (Figure 2). In addition, the expression of IAPP is highly correlated with early and late spermatids (corr: 0.678 and 0.619, respectively) within testis. These predictions are also in agreement with data from other sections in the HPA database. IAPP is classified as enriched in the pancreas and group enriched in pancreatic endocrine cells (i.e., cells in the islets of Langerhans) and early spermatids in the Tissue and Single Cell Type sections, respectively. The expression of IAPP within islets of Langerhans is also validated on the protein level using IHC, showing cytoplasmic expression within a subset of the cells within the islets of Langerhans.

Tissue samples used for omics analysis are collected from various parts of human organs and tissues. Since the three‐dimensional architecture of tissues varies at different locations, this can give rise to significant between‐sample variation in terms of cell type proportions and gene expression. Skin has a particularly complex tissue architecture involving several layers found at different depths, each with its distinct features. Some of the cellular structures that are not commonly found in the skin samples analyzed with IHC include hair root structures, sebaceous glands, and eccrine sweat glands. Eccrine sweat glands consist of a secretory coil found in the deep part of the dermis layer of the skin that is connected to a single tube that leads the sweat from the secretory coils to an opening at the skin surface (Cui & Schlessinger, 2015). Engrailed homeobox 1 (EN1) belongs to the group of 61 genes classified as very highly enriched in eccrine sweat gland cells (corr: 0.761) according to the Tissue Cell Type section (Figure 2). Expression levels from RNA‐seq data at tissue and single cell level indicate some specificity linked to skin and melanocytes, respectively, but do not provide any clear indication of expression within the cells of eccrine sweat glands. However, extended in‐depth characterization of selected areas of skin containing structures previously not stained using IHC show clear nuclear staining in eccrine sweat gland cells, illustrating the benefit of an ongoing effort within the HPA program to expand and deepen the antibody‐based exploration of human tissues.

### 2.3 High‐resolution spatial proteomics

The major collection of bioimages in the HPA database, comprising 10 million high‐resolution images of stained tissues and cells, constitutes an important resource for studying protein expression in relation to tissue and cell morphology. While the staining of the most common cell types and structures have been manually annotated and is presented as protein expression levels, each image contains much more information regarding the exact spatial localization, including expression in subsets of cells and rare cell types. In an effort to increase the resolution of the tissue‐based protein expression data, relevant sets of genes, mostly comprising genes that based on mRNA expression have been shown to be elevated in specific tissues or cell types, have undergone an in‐depth characterization. By involving international experts with extensive knowledge regarding certain human organs, the spatial data could be analyzed with a higher resolution. Available images of human testis served as a first pilot project to distinguish protein expression in eight different cell types instead of previously two (Ghoshal et al., 2021; Pineau et al., 2019). Due to the complex physiological processes taking place during spermatogenesis and the fact that testis harbors a large number of proteins not previously characterized, this organ is a priority for ongoing follow‐up studies related to the Tissue section (Green et al., 2018; Guo et al., 2018). Based on a similar strategy, a detailed characterization was performed for cerebellum, lung, bronchus, nasopharynx, fallopian tube, placenta, kidney, duodenum, colon, rectum, small intestine, and two different skin samples. In total, 7260 genes have undergone an in‐depth characterization of existing images in the HPA database, resulting in novel datasets with detailed protein expression data in 63 new cell types from 14 different organs. The new data constitutes an excellent resource for comparison with other single cell efforts, including the information generated as part of the Single Cell Type and Tissue Cell Type sections, or various external initiatives mapping the human body at a single cell level.

### 2.4 Other recent updates to the HPA database

In addition to the Single Cell Type and Tissue Cell Type sections, two other novel sections have been introduced in version 21: the Blood Protein and the Cell Line sections. The *Blood Protein* section refers to datasets related to the plasma proteome and the human secretome, that were previously included in the Blood Atlas section (Digre & Lindskog, 2021). In version 21, plasma concentrations based on mass spectrometry data from a new build of the Peptide Atlas are presented for 4072 secreted and leakage proteins. Plasma levels for 1456 proteins measured in a 2‐year longitudinal study of healthy individuals using proximity extension assay combined with next generation DNA sequencing (Olink Explore) are also presented (Tebani et al., 2020). The data summarized as the human secretome, that is, the collection of proteins expected to be secreted (Uhlen et al., 2019a), has been updated and a new category, Immunoglobulin genes, has been added. The total number of predicted secreted proteins is now 2739 and the number of proteins annotated as secreted to blood is 784. These proteins constitute a particularly interesting group of proteins for diagnostic and drug development purposes. Plasma concentrations for 435 proteins predicted to be secreted to blood have been collected from immunoassay based studies in literature and are displayed together with information on their predicted function. The transcriptomics data from 18 different immune cell types separated by flow sorting that was previously included in the Blood Atlas (Uhlen et al., 2019b) are now presented under the *Immune Cell* section.

The *Cell Line* section is based on the cell line expression data that was earlier part of the Cell Atlas. The previous data on subcellular localization of human proteins using cell lines (Thul et al., 2017) can now be found under the *Subcellular* section. In the Subcellular section, a new normalization strategy for staining intensities of cell cycle dependent markers was used, leading to the definition of an additional 129 genes with cell cycle dependent transcripts (Mahdessian et al., 2021).

In the *Brain* section, the previous transcriptomics data based on GTEx and FANTOM5 was replaced by internally generated RNA‐seq data from 967 human brain samples including >200 regions, areas and nuclei of the human brain. No new updates were made to the *Pathology* and *Metabolic* sections that have not been described previously (Robinson et al., 2020; Sivertsson et al., 2020; Uhlen et al., 2017). It should however be noted that the HPA database is built upon protein‐coding genes as defined by Ensembl, which means that each time the HPA links the data to a new version of Ensembl (currently Ensembl version 103.38), numbers of genes and proteins analyzed with different assays as well as all gene specificity classifications will be affected. Furthermore, the protein expression data in the Tissue, Pathology and Subcellular sections is constantly being reviewed to ensure antibody specificity, and antibody stainings may be replaced or removed between versions if novel information suggests that the data is contradictory. A full list of such antibody changes, as well as information on the major updates for each version of the HPA database can be found under the Release history (https://www.proteinatlas.org/about/releases).

## 3 BIG DATA ORGANIZATION AND VISUALIZATION

The HPA online resource has expanded rapidly in recent years with additions of several large‐scale analyses of transcriptomic datasets at both tissue and single cell resolution to support and expand the already existing antibody‐based exploration of proteins, involving all parts of the human body, healthy and cancer tissues, as well as cell lines, complemented by studies made on tissues from pig and mouse. The assembly of such extensive amounts of multi‐dimensional data from integrated omics technologies makes it difficult to utilize and interpret. Hence, a lot of effort has been put into creating a user‐friendly website structure with easy‐to‐interpret graphics to guide the visitor through the data and highlight important aspects of the information. The various types of data in the HPA website have recently been organized into 10 sections to simplify the exploration of proteins. Further, the section‐based structure of the website is designed to allow the visitor three different ways to make use of the data: (i) *browse through expression profiles* of the various categories of each section, such as the expression of genes in different organs, tissues, cell types, cell lines or subcellular compartments, and also explore the proteins involved in different pathways of human metabolism, (ii) *identify and study the expression of individual genes or proteins* through the various perspectives of the 10 sections using the advanced search engine on the website with built in filters to discriminate according to various categories and features associated with the gene or protein at the HPA, or (iii) *download the data* in raw or processed form for free analysis and exploration.

Holistic computational analysis of large transcriptomic datasets and subsequent visualization and interpretation of the resulting data require advanced mathematical models. After the transcript count data has been processed to allow for sample comparison and clustered to map the relationship between all data points, the data will include a large number of dimensions that cannot be plotted in a single graph. To solve this problem, dimensionality reduction methods are used to create two‐dimensional representations of the data that can then be plotted in a normal graph. A commonly used method for dimensionality reduction of high‐dimensional data is the uniform manifold approximation and projection (UMAP) method. UMAP representations of the data create a two‐dimensional landscape of data points that are divided into different clusters based on the distances between the data points, where neighboring data points that are close to each other and form clusters have similar expression patterns and vice versa. Thus, data points within a cluster often share some trait, like cells of the same cell type or genes that are part of the same biological process.

UMAP analysis was vital in the creation of the Single Cell Type section of HPA (Karlsson et al., 2021). Since cells of the same cell type have similar expression profiles compared to other types of cells, they cluster together in the UMAP analysis of scRNA‐seq data, forming the basis for identifying cell type clusters. In addition to analysis of single cell type expression across genes, UMAP was also employed in an opposite fashion to explore the relationship between all protein‐coding genes across tissues, cell types, immune cells, and cell lines, as a help to uncover previously unknown associations between genes (Karlsson et al., 2022). This new way of expression clustering constitutes a novel classification of all protein‐coding genes. The other classification system entails stratification of genes according to the specificity and distribution of their expression in various tissues, cell types or cell lines, which is limited to genes that are detected (>1.0 nTPM) or have relatively high expression in one or few tissues, cell types or cell lines. Thus, there are risks of neglecting genes with semi‐ubiquitous expression patterns across tissues and cells that fall outside specific cutoffs. By stratifying genes according to their expression pattern across tissues and cells, genes that were previously neglected can be picked up based on their association with a gene cluster containing groups of genes involved in well‐known processes.

Each of the gene clusters identified in the UMAP expression analysis were manually assigned with a main specificity and function based on summary analyses of the expression specificity and other protein characterization data associated with the genes within the clusters. The gene cluster UMAP visualizations revealed close association between many of the clusters annotated as non‐specific or with unknown or basic cellular functions, such as “transcription”, “mitochondria”, and “cell cycle regulation”. Most of these clusters are located close to the center area of the UMAPs. Other sets of gene clusters that were found grouped together in the different UMAPs include clusters related to the immune system, nervous system and spermatogenesis. Overall, the UMAPs displayed a pattern of close association between clusters, except for the cell line version that showed a high degree of cluster dispersion. This could possibly be a result of the specialization of expression seen for established in vitro grown cell lines, while the “boundaries” between the expression of different tissues and cell types are less pronounced (Uhlen et al., 2019b).

**FIGURE 3:**
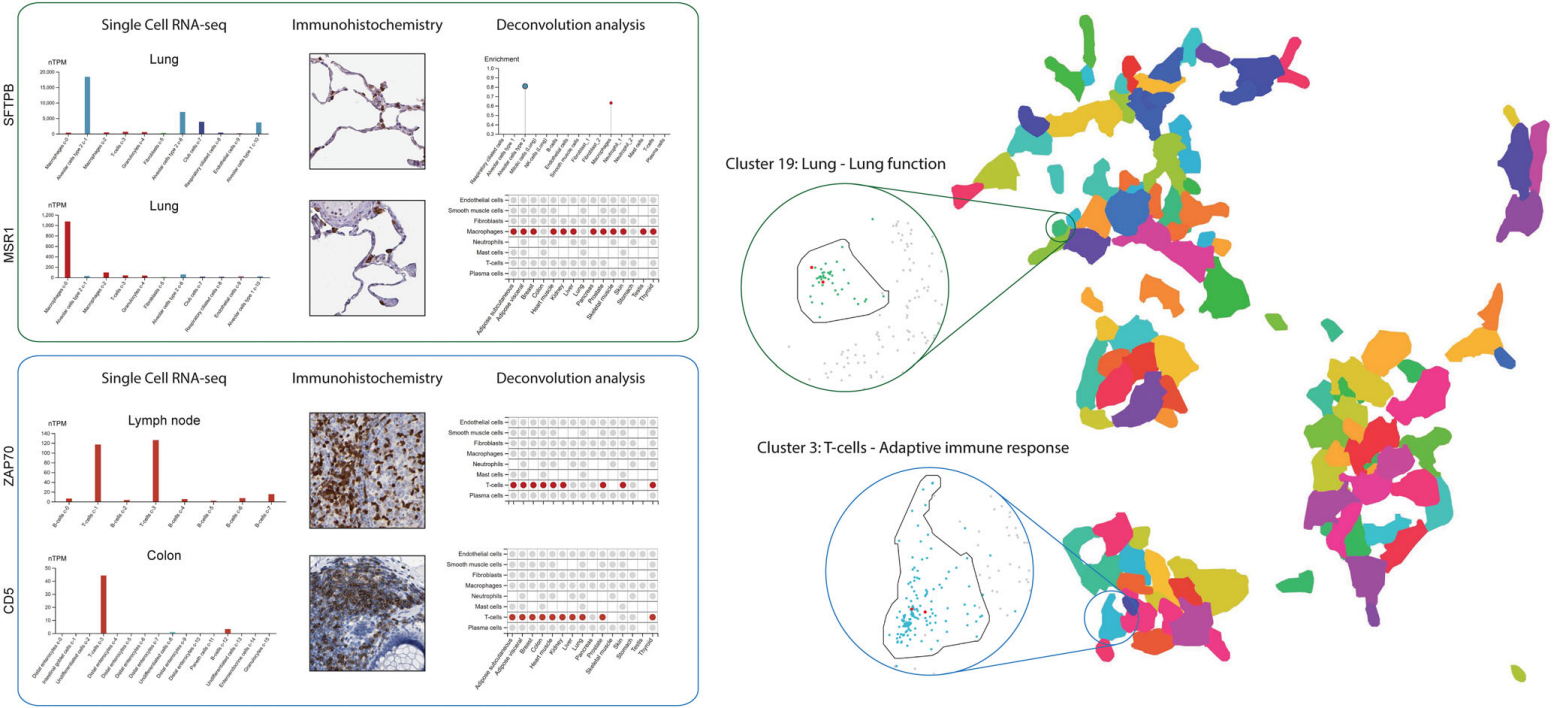
Visualization of gene association through expression clustering. UMAP visualization of all gene expression clusters based on the Tissue section bulk RNA‐seq data along with examples of members in cluster 3 and 19. For each protein example, gene expression data at single cell resolution (single cell RNA‐seq, immunohistochemistry and deconvolution analysis) are shown for their relevant tissue. Single cell RNA‐seq data is shown as normalized transcripts per million (nTPM) in tissue‐specific bar charts. Deconvolution analysis is represented by a plot with enrichment scores for each cell type above cutoff (0.3) for SFTPB and core cell type tables, indicating if the expression specificity for a gene in a cell type is enriched (red dot), not enriched (gray dot) or not present (no dot) in a tissue, for MSR1, ZAP70, and CD5. MSR1, Macrophage scavenger receptor 1; SFTPB, surfactant protein B

In order to evaluate the utility of the gene clustering, individual clusters within the Tissue version were explored to find interesting examples of associated genes (Figure 3). Cluster 19 is a relatively small cluster of 44 genes annotated as mainly lung‐specific, both in terms of expression and function. The lung is made up to a large extent of alveoli, air sacs where oxygen is exchanged for carbon dioxide in the blood. The alveoli include two types of alveolar cell types, type 1 and type 2, that form the air sac structure and produce surfactant in order to lower the surface tension in the fluids that cover the inner surface of the alveoli, respectively. Proteins make up about 10% of the surfactant mixture and one of these proteins is the cluster 19 member surfactant protein B (SFTPB). SFTPB has enriched expression in lung and type 2 alveolar cells according to the Tissue, Single Cell Type and Tissue Cell Type sections, as well as being clearly localized in type 2 alveolar cells in IHC‐stained lung tissue. The alveoli are patrolled by resident scavenging macrophages that clear away foreign particles and damaged cells. Macrophage scavenger receptor 1 (MSR1), another member of cluster 19, is a cell surface receptor that is able to engulf macromolecules through endocytosis. The expression of MSR1 shows enrichment in various types of macrophages throughout the body according to the sections with single cell resolution data in the HPA. Interestingly, there is especially high expression in lung according to the Tissue section and in one of two macrophage cell type clusters in the Single Cell Type section. Since lung is known to harbor at least two types of macrophages, alveolar, and interstitial macrophages (Hou et al., 2021), the divergent expression suggests that the expression of MSR1 is mostly limited to one of the types.

Cluster 3 is annotated as mainly T‐cell‐specific with adaptive immune response as the main function. It is part of a separated group of immune‐clusters made up to a large extent by immune system‐related genes. T‐cell receptor (TCR) signaling plays a central role in T‐cell function by regulating the maturation and effector functions of T‐cells. Cluster 3 includes many genes that are associated with TCR signaling, including co‐receptor CD5 and the downstream signaling protein ZAP70 (Matson et al., 2020). Both genes exhibit elevated expression in lymphoid tissues, bone marrow and T‐cells according to the various sections at the HPA. Additionally, distinct IHC‐based protein expression is seen in lymphoid tissue‐associated immune cells, known to be rich in T‐cells.

## 4 DISCUSSION

The HPA is a knowledge resource for studying various aspects of human proteins. Since its first launch in 2005, it has grown into one of the most visited biological databases in the world with more than 400,000 visitors per month both from industry and academia. The HPA program has already contributed to tens of thousands of publications in the field of human biology and disease, emphasizing its importance for the life science community. The consortium has in addition to the in‐house generated HPA antibodies also tested >10,000 external antibodies from various commercial vendors or research institutes. One such example is the National Cancer Institute's Clinical Proteomic Tumor Analysis Consortium (CPTAC), which is an effort to accelerate the understanding of the molecular basis of cancer through the application of large‐scale proteome and genome analysis, or proteogenomics. A large number of the antibodies available via CPTAC's Antibody Portal (Edwards et al., 2015) have been validated by the standardized quality assurance workflows by the HPA project, and the HPA has thus made an important contribution to studies using these antibodies. This collaboration constitutes an example of the importance of stringent antibody validation for reliable biomarker studies.

Here, we summarized recent updates in data and structure of the HPA database, showing that the HPA has taken one step further towards “big data” and integration of various omics technologies, by utilizing both scRNA‐seq and standardized immunohistochemistry to measure the expression of human transcripts and proteins in single cell types. Several efforts worldwide focus on the generation of single‐cell mapping of human tissues in health and disease. Most of these efforts focus on scRNA‐seq only, and since proteins are the main molecules carrying out the functions of human cells and constitute the targets for most pharmaceutical drugs, studying proteins is crucial for future precision medicine efforts.

While immunohistochemistry is the main method for studying proteins with a spatial localization, mass spectrometry imaging (MSI) methods such as matrix‐assisted laser desorption/ionization (MALDI) (Buchberger et al., 2018) offers a possibility of quantitative measurements. Several different ionization methods exist, each with its advantages and disadvantages, but presently, few methods are compatible with paraffin‐embedded tissue sections or require specially coated slides, thus limiting the applicability on available biobank material that are directly comparable with antibody‐based imaging data. Furthermore, MSI does not offer single cell resolution. Nevertheless, the HPA project actively follows further advancements in various MSI technologies to determine if inclusion of such data in the HPA portal would be feasible in the near future. Another emerging effort that allows for increased single cell resolution includes a novel technology called Deep Visual Proteomics (DVP) (Mund et al., 2022). The DVP technology combines sub‐micron‐resolution imaging, single‐cell phenotyping based on artificial intelligence (AI) and isolation with an ultra‐sensitive proteomics workflow, and constitutes an interesting approach likely to serve as a complement to spatial proteomics and standard mass spectrometry in the near future. The field of multiplexed imaging is rapidly growing, allowing for detection of multiple proteins in the same tissue section which enables molecular insights into co‐expression and tissue heterogeneity. Also in the transcriptomics field, major method development is ongoing, and several powerful technologies have been established to overcome the loss of transcript location in relation to the tissue geography. The two most common methods are spatial transcriptomics (Andersson et al., 2020) and in situ hybridization (ISH) using RNAScope™ (Maynard et al., 2021). While several research projects that involved integrating various methods for mRNA and protein detection have been published, most focused on a single tissue or a certain set of genes, and no other effort than the HPA combines transcriptomics and proteomics at a large scale to validate data across disciplines. For a full understanding of the human building blocks and determining the function of each human protein, it is necessary to take into consideration the specific characteristics of each method and dataset.

It is likely that future versions of the HPA database will include integration of novel technologies for mRNA and protein detection, increasing the width and depth of the data for studying proteins at different levels.

Nevertheless, the current datasets constitute a major resource for studying human biology in health and disease, both from the perspective of single genes, tissues or cell types, as well as from a body‐wide or genome‐wide perspective, taking advantage of the publically available knowledge resource. Further, the major image bank of >10 million high‐resolution images of both normal and cancer tissues that all have been manually annotated by experts forms an important collection of labeled images for AI and machine learning efforts, forming a first step towards novel insights on human physiology in health and disease.

## AUTHOR CONTRIBUTIONS

**Andreas Digre:** Formal analysis (lead); investigation (lead); visualization (equal); writing – original draft (equal); writing – review and editing (equal). **Cecilia Lindskog:** Conceptualization (lead); project administration (lead); resources (lead); supervision (lead); validation (lead); visualization (equal); writing – original draft (equal); writing – review and editing (equal).

## CONFLICTS OF INTEREST

The authors declare no conflicts of interest.

## Data Availability

All data presented in the manuscript is publicly available at https://www.proteinatlas.org.

## References

[pro4562-bib-0001] Adhikari S , Nice EC , Deutsch EW , Lane L , Omenn GS , Pennington SR , et al. A high‐stringency blueprint of the human proteome. Nat Commun. 2020;11(1):1–16. 33067450 10.1038/s41467-020-19045-9 PMC7568584

[pro4562-bib-0002] Andersson A , Bergenstråhle J , Asp M , Bergenstråhle L , Jurek A , Navarro JF , et al. Single‐cell and spatial transcriptomics enables probabilistic inference of cell type topography. Commun Biol. 2020;3(1):1–8. 33037292 10.1038/s42003-020-01247-y PMC7547664

[pro4562-bib-0003] Buchberger AR , DeLaney K , Johnson J , Li L . Mass spectrometry imaging: a review of emerging advancements and future insights. Anal Chem. 2018;90(1):240–65. 29155564 10.1021/acs.analchem.7b04733 PMC5959842

[pro4562-bib-0004] Butler LM , Hallstrom BM , Fagerberg L , Ponten F , Uhlen M , Renne T , et al. Analysis of body‐wide unfractionated tissue data to identify a core human endothelial transcriptome. Cell Syst. 2016;3(3):287–301 e3. 27641958 10.1016/j.cels.2016.08.001

[pro4562-bib-0005] Chen G , Ning B , Shi T . Single‐cell RNA‐Seq technologies and related computational data analysis. Front Genet. 2019;10:317. 31024627 10.3389/fgene.2019.00317 PMC6460256

[pro4562-bib-0006] Consortium H . The human body at cellular resolution: the NIH human biomolecular atlas program. Nature. 2019;574(7777):187–92. 31597973 10.1038/s41586-019-1629-x PMC6800388

[pro4562-bib-0007] Cui CY , Schlessinger D . Eccrine sweat gland development and sweat secretion. Exp Dermatol. 2015;24(9):644–50. 26014472 10.1111/exd.12773 PMC5508982

[pro4562-bib-0008] Digre A , Lindskog C . The human protein atlas‐spatial localization of the human proteome in health and disease. Protein Sci. 2021;30(1):218–33. 33146890 10.1002/pro.3987 PMC7737765

[pro4562-bib-0009] Dusart P , Hallstrom BM , Renne T , Odeberg J , Uhlen M , Butler LM . A systems‐based map of human brain cell‐type enriched genes and malignancy‐associated endothelial changes. Cell Rep. 2019;29(6):1690–1706 e4. 31693905 10.1016/j.celrep.2019.09.088

[pro4562-bib-0010] Edwards NJ , Oberti M , Thangudu RR , Cai S , McGarvey PB , Jacob S , et al. The CPTAC data portal: a resource for cancer proteomics research. J Proteome Res. 2015;14(6):2707–13. 25873244 10.1021/pr501254j

[pro4562-bib-0011] Ghoshal B , Hikmet F , Pineau C , Tucker A , Lindskog C . DeepHistoClass: a novel strategy for confident classification of immunohistochemistry images using deep learning. Mol Cell Proteomics. 2021;20:100140. 34425263 10.1016/j.mcpro.2021.100140 PMC8476775

[pro4562-bib-0012] Green CD , Ma Q , Manske GL , Shami AN , Zheng X , Marini S , et al. A comprehensive roadmap of murine spermatogenesis defined by single‐cell RNA‐Seq. Dev Cell. 2018;46(5):651–667e10. 30146481 10.1016/j.devcel.2018.07.025 PMC6713459

[pro4562-bib-0013] Guo J , Grow EJ , Mlcochova H , Maher GJ , Lindskog C , Nie X , et al. The adult human testis transcriptional cell atlas. Cell Res. 2018;28(12):1141–57. 30315278 10.1038/s41422-018-0099-2 PMC6274646

[pro4562-bib-0014] Han X , Zhou Z , Fei L , Sun H , Wang R , Chen Y , et al. Construction of a human cell landscape at single‐cell level. Nature. 2020;581(7808):303–9. 32214235 10.1038/s41586-020-2157-4

[pro4562-bib-0015] Hikmet F , Méar L , Edvinsson Å , Micke P , Uhlén M , Lindskog C . The protein expression profile of ACE2 in human tissues. Mol Syst Biol. 2020;16(7):e9610. 32715618 10.15252/msb.20209610 PMC7383091

[pro4562-bib-0016] Hou F , Xiao K , Tang L , Xie L . Diversity of macrophages in lung homeostasis and diseases. Front Immunol. 2021;12:753940. 34630433 10.3389/fimmu.2021.753940 PMC8500393

[pro4562-bib-0017] Karlsson M , Zhang C , Méar L , Zhong W , Digre A , Katona B , et al. A single‐cell type transcriptomics map of human tissues. Sci Adv. 2021;7(31):eabh2169. 34321199 10.1126/sciadv.abh2169 PMC8318366

[pro4562-bib-0018] Karlsson M , Shi M , Méar L , Schutten R , Hikmet F , Digre A , et al. Genome‐wide single cell annotation of the human protein‐coding genes. 2022. httpss://doi.org/10.1101/2022.08.03.502627.

[pro4562-bib-0019] Keen JC , Moore HM . The genotype‐tissue expression (GTEx) project: linking clinical data with molecular analysis to advance personalized medicine. J Pers Med. 2015;5(1):22–9. 25809799 10.3390/jpm5010022 PMC4384056

[pro4562-bib-0020] Mahdessian D , Cesnik AJ , Gnann C , Danielsson F , Stenstrom L , Arif M , et al. Spatiotemporal dissection of the cell cycle with single‐cell proteogenomics. Nature. 2021;590(7847):649–54. 33627808 10.1038/s41586-021-03232-9

[pro4562-bib-0021] Matson CA , Choi S , Livak F , Zhao B , Mitra A , Love PE , et al. CD5 dynamically calibrates basal NF‐kappaB signaling in T cells during thymic development and peripheral activation. Proc Natl Acad Sci U S A. 2020;117(25):14342–53. 32513716 10.1073/pnas.1922525117 PMC7322041

[pro4562-bib-0022] Maynard KR , Collado‐Torres L , Weber LM , Uytingco C , Barry BK , Williams SR , et al. Transcriptome‐scale spatial gene expression in the human dorsolateral prefrontal cortex. Nat Neurosci. 2021;24:425–36. 33558695 10.1038/s41593-020-00787-0 PMC8095368

[pro4562-bib-0023] Mund A , Coscia F , Kriston A , Hollandi R , Kovacs F , Brunner AD , et al. Deep visual proteomics defines single‐cell identity and heterogeneity. Nat Biotechnol. 2022;40(8):1231–40. 35590073 10.1038/s41587-022-01302-5 PMC9371970

[pro4562-bib-0024] Pineau C , Hikmet F , Zhang C , Oksvold P , Chen S , Fagerberg L , et al. Cell type‐specific expression of testis elevated genes based on transcriptomics and antibody‐based proteomics. J Proteome Res. 2019;18(12):4215–30. 31429579 10.1021/acs.jproteome.9b00351

[pro4562-bib-0025] Refaeli I , Hughes MR , Wong AK , Bissonnette MLZ , Roskelley CD , Wayne Vogl A , et al. Distinct functional requirements for podocalyxin in immature and mature podocytes reveal mechanisms of human kidney disease. Sci Rep. 2020;10(1):9419. 32523052 10.1038/s41598-020-64907-3 PMC7286918

[pro4562-bib-0026] Regev A , Teichmann SA , Lander ES , Amit I , Benoist C , Birney E , et al. The human cell atlas. Elife. 2017;6:1–30. 10.7554/eLife.27041 PMC5762154 29206104

[pro4562-bib-0027] Reiser J , Altintas MM . Podocytes. F1000Res. 2016;5:5. 10.12688/f1000research.7255.1 PMC4755401 26918173

[pro4562-bib-0028] Robinson JL , Kocabas P , Wang H , Cholley PE , Cook D , Nilsson A , et al. An atlas of human metabolism. Sci Signal. 2020;13(624):1–11. 10.1126/scisignal.aaz1482 PMC7331181 32209698

[pro4562-bib-0029] Sivertsson A , Lindstrom E , Oksvold P , Katona B , Hikmet F , Vuu J , et al. Enhanced validation of antibodies enables the discovery of missing proteins. J Proteome Res. 2020;19(12):4766–81. 33170010 10.1021/acs.jproteome.0c00486 PMC7723238

[pro4562-bib-0030] Tabula Sapiens C , Jones RC , Karkanias J , Krasnow MA , Pisco AO , Quake SR , et al. The Tabula Sapiens: a multiple‐organ, single‐cell transcriptomic atlas of humans. Science. 2022;376(6594):eabl4896. 35549404 10.1126/science.abl4896 PMC9812260

[pro4562-bib-0031] Tebani A , Gummesson A , Zhong W , Koistinen IS , Lakshmikanth T , Olsson LM , et al. Integration of molecular profiles in a longitudinal wellness profiling cohort. Nat Commun. 2020;11(1):4487. 32900998 10.1038/s41467-020-18148-7 PMC7479148

[pro4562-bib-0032] Thul PJ , Akesson L , Wiking M , Mahdessian D , Geladaki A , Ait Blal H , et al. A subcellular map of the human proteome. Science. 2017;356(6340):1–13. 10.1126/science.aal3321 28495876

[pro4562-bib-0033] Uhlen M , Fagerberg L , Hallstrom BM , Lindskog C , Oksvold P , Mardinoglu A , et al. Proteomics. Tissue‐based map of the human proteome. Science. 2015;347(6220):1260419. 25613900 10.1126/science.1260419

[pro4562-bib-0034] Uhlen M , Zhang C , Lee S , Sjostedt E , Fagerberg L , Bidkhori G , et al. A pathology atlas of the human cancer transcriptome. Science. 2017;357(6352):1–13. 10.1126/science.aan2507 28818916

[pro4562-bib-0035] Uhlen M , Karlsson MJ , Hober A , Svensson AS , Scheffel J , Kotol D , et al. The human secretome. Sci Signal. 2019a;12:609. 10.1126/scisignal.aaz0274 31772123

[pro4562-bib-0036] Uhlen M , Karlsson MJ , Zhong W , Tebani A , Pou C , Mikes J , et al. A genome‐wide transcriptomic analysis of protein‐coding genes in human blood cells. Science. 2019b;366(6472):1–13. 10.1126/science.aax9198 31857451

[pro4562-bib-0037] Wang D , Eraslan B , Wieland T , Hallstrom B , Hopf T , Zolg DP , et al. A deep proteome and transcriptome abundance atlas of 29 healthy human tissues. Mol Syst Biol. 2019;15(2):e8503. 30777892 10.15252/msb.20188503 PMC6379049

[pro4562-bib-0038] Yu NY , Hallstrom BM , Fagerberg L , Ponten F , Kawaji H , Carninci P , et al. Complementing tissue characterization by integrating transcriptome profiling from the human protein atlas and from the FANTOM5 consortium. Nucleic Acids Res. 2015;43(14):6787–98. 26117540 10.1093/nar/gkv608 PMC4538815

